# Exploring cognitive trajectories and their association with physical performance: evidence from the China Health and Retirement Longitudinal Study

**DOI:** 10.4178/epih.e2023064

**Published:** 2023-07-09

**Authors:** Jingdong Suo, Xianlei Shen, Jinyu He, Haoran Sun, Yu Shi, Rongxin He, Xiao Zhang, Xijie Wang, Yuandi Xi, Wannian Liang

**Affiliations:** 1 Vanke School of Public Health, Tsinghua University, Beijing, China; 2 Institute for Healthy China, Tsinghua University, Beijing, China; 3 Beijing Key Laboratory of Environmental Toxicology, School of Public Health, Capital Medical University, Beijing, China

**Keywords:** Cognitive aging, Physical performance, China Health and Retirement Longitudinal Study, Longitudinal study

## Abstract

**OBJECTIVES:**

The long-term trends of cognitive function and its associations with physical performance remain unclear, particularly in Asian populations. The study objectives were to determine cognitive trajectories in middle-aged and elderly Chinese individuals, as well as to examine differences in physical performance across cognitive trajectory groups.

**METHODS:**

Data were extracted from the China Health and Retirement Longitudinal Study. A total of 5,701 participants (47.7% male) with a mean age of 57.8 (standard deviation, 8.4) years at enrollment were included. A group-based trajectory model was used to identify cognitive trajectory groups for each sex. Grip strength, repeated chair stand, and standing balance tests were used to evaluate physical performance. An ordered logistic regression model was employed to analyze differences in physical performance across cognitive trajectory groups.

**RESULTS:**

Three cognitive trajectory groups were identified for each sex: low, middle, and high. For both sexes, higher cognitive trajectory groups exhibited smaller declines with age. In the fully adjusted model, relative to the low trajectory group, the odds ratios (ORs) of better physical performance in the middle cognitive group were 1.37 (95% confidence interval [CI], 1.17 to 1.59; p<0.001) during follow-up and 1.40 (95% CI, 1.20 to 1.64; p<0.001) at the endpoint. The ORs in the high trajectory group were 1.94 (95% CI, 1.61 to 2.32; p<0.001) during follow-up and 2.04 (95% CI, 1.69 to 2.45; p<0.001) at the endpoint.

**CONCLUSIONS:**

Cognitive function was better preserved in male participants and individuals with higher baseline cognitive function. A higher cognitive trajectory was associated with better physical performance over time.

## INTRODUCTION

The global population and life expectancy are growing, leading to an escalating aging problem. In 2018, 249 million individuals in China were aged 60 and older, accounting for 17.9% of the total population [1]. By 2050, this population is projected to exceed 450 million, representing a share of over 30% [2]. However, the increase in healthy life expectancy has been much slower than that of overall life expectancy [3], resulting in a rapid rise in age-related diseases.

A growing public health concern, aging is associated with declines in bodily functions and physical performance [4]. Previous studies have demonstrated that a decrease in physical performance often marks the early stages of disability in older individuals [5]. Poor physical performance has been consistently linked to adverse health risks, including total and cause-specific mortality [6], depression [7], falls [8], frailty [9], and hospitalization [10]. However, the indicators of physical performance vary across studies and can include grip strength, standing balance, gait speed, functional reach tests, and chair stands. Although it is widely accepted that maintaining good physical performance in old age is important, further research is required to better understand its longitudinal and overall development.

Cognitive impairment is an early stage of dementia, determined by a wide variety of neurological, psychological, and emotional factors [11,12]. The rapid aging process increases the burden of age-related cognitive decline, which occurs in many areas, including memory storage, emotional control, processing speed, and decision-making [12]. Currently, 50 million people worldwide are estimated to have dementia, and this number is expected to reach 81.1 million by 2040 [13]. Rather than relying on single observations, long-term follow-up and identification of cognitive trajectory constitute approaches for investigating cognitive changes over time, along with the determinants of such changes [14]. However, since relatively few studies have examined the cognitive trajectories of middle-aged and elderly people, the population-based phenotypes of cognitive change remain unclear.

Recently, studies have demonstrated that changes in cognitive function are associated with physical performance in the areas of mobility [15], balance [16], and muscle strength [17]. However, these findings primarily stem from single-center, cross-sectional studies with relatively small sample sizes, and they vary depending on the study design [15,18]. Consequently, we sought to investigate the progression of cognitive function over time and its association with physical performance using a multicenter, nationally representative cohort in China.

Utilizing data from the 2011, 2013, and 2015 waves of the China Health and Retirement Longitudinal Study (CHARLS), this study was conducted with the goal of identifying diverse patterns of cognitive trajectories and examining their associations with physical performance outcomes in middle-aged and elderly populations.

## MATERIALS AND METHODS

### Study participants

The data for this study was obtained from the CHARLS, a nationally representative prospective cohort survey conducted by the National School of Development at Peking University. The baseline survey took place in 2011 and was followed by 2 additional surveys in 2013 and 2015, utilizing a multistage probability sampling design and a probability-proportional-to-size sampling technique [19]. The CHARLS included thousands of participants aged 45 years and older from 150 counties/districts and 450 villages/resident committees across 28 provinces in China, with a large sample size, strong representation, and high data quality. Personal information, mental and physical health data, and basic family information were collected during each wave of the survey.

In the present study, participants who completed all 3 waves of surveying were included (7,697 of 16,381; 47.0%). Among these individuals, those with incomplete demographic information (n=36), missing physical performance indicators (no responses for all 4 measurements, n=891), and incomplete cognitive function measurements (missing more than 6 items, n=1,069) were excluded. Consequently, 5,701 participants were included in the data analysis.

### Physical performance measurement

Physical performance was considered to be indicated by grip strength, repeated chair stands, and 2 standing balance tests. Each of these indicators was assigned a score of either 0 or 1, depending on whether they were completed successfully. The total physical performance score was calculated by summing the scores of the 3 indicators, and this was done at baseline, during follow-up, and at the endpoint. The physical performance score ranged from 0 to 3.

#### Grip strength

Grip strength, an indicator of upper limb muscle strength, was measured using a dynamometer (Yuejian WL-1000; Nantong Yuejian Physical Measurement Instrument Co., Ltd., Nantong, China) and recorded in kilograms [6,19]. During testing, participants were asked to stand (unless physically precluded from doing so), hold the dynamometer at a right angle, squeeze the handle as hard as possible for a few seconds, and then release. Grip strength for each hand was tested twice in an alternating manner, and the average of the 4 tests was recorded [20]. For 556 participants with 1 missing test, the average of the other completed tests was used for the analysis. Participants were assigned 1 point if the score was above the median and 0 points if it was not.

#### Repeated chair stands

Repeated chair stands, an indicator of lower limb muscle strength, were assessed using a standard chair with a height of 47 centimeters from the floor [21]. Participants were instructed to sit in the chair with their arms folded across their chest, then to stand up straight and sit back down as quickly as possible 5 times without stopping or using their arms for assistance. A score of 1 point was given if the participant successfully completed the test, and a score of 0 points was given otherwise.

#### Standing balance test

The standing balance test, which evaluates static balance, endurance, and postural control ability, was assessed using semitandem stands and full-tandem stands [22]. In the semi-tandem stand test, participants were required to stand with the side of the heel of one foot touching the big toe of the other foot for 10 seconds without losing balance, moving the feet, or touching anything. For the full-tandem stand test, participants were instructed to stand with the heel of one foot in front of and touching the toes of the other foot. A 30-second full-tandem stand test was used for participants aged 70 years or older, while a 60-second full-tandem stand test was used for those under 70 years old. Participants were awarded 1 point if they successfully completed both tests and 0 points if they did not.

### Cognitive assessment

Cognitive function in the CHARLS was assessed across 2 dimensions, evaluating orientation, attention, visuospatial abilities, and word recall [23,24]. The mental status dimension incorporated information from the Telephone Interview of Cognitive Status, which included orientation, numeric ability, and visuospatial abilities. Orientation was assessed by asking respondents to provide the date, season, and day of the week (5 points). Numeric ability was measured by having respondents subtract 7 from 100 five consecutive times (5 points). Visuospatial abilities were evaluated by presenting respondents with a picture and asking them to redraw the image just shown (1 point). The episodic memory dimension involved collecting data on immediate word recall (10 points) and delayed word recall (10 points). The total cognitive function score was calculated by summing the scores from each dimension, with a possible range of 0 points (worst) to 31 points (best).

### Covariates

Baseline socio-demographic characteristics and health-related factors were considered for the adjusted analysis. Socio-demographic characteristics included age, sex (male or female), residence (urban or rural), province (of 28 provinces), marital status (married or not), and education level (illiterate, elementary school, or middle school and above). Residence was defined based on China’s National Bureau of Statistics, with urban areas including cities, city suburbs, towns, town suburbs, and other special zones, while all other areas were considered rural [19,25]. For marital status, individuals were considered married if they were either living with their spouse or temporarily separated due to reasons such as work. Regarding education level, respondents were classified as illiterate if they had no formal education or did not complete primary school, while all others were categorized as having completed elementary school or higher. Health-related factors included body mass index (BMI), smoking status (yes/no), drinking status (yes/no), and diagnosis of chronic non-communicable diseases (yes/no).

### Statistical analysis

We utilized group-based trajectory modeling to identify distinct trajectory groups of cognitive function as they relate to age [27]. Trajectories were modeled using a censored normal distribution by sex, with age in years serving as the measure of time. The bestfit model was determined based on the following 4 criteria: (1) the Bayesian information criterion of the alternative model decreased by at least 20 relative to the previous model; (2) the odds of correct classification, a measure of assignment accuracy, exceeded 5; (3) the average posterior probability for each group was greater than 0.7; and (4) the sample size of each group constituted at least 5% of the total. Given that cognitive trajectories may differ by sex, we conducted sex-specific modeling. Initially, we modeled adherence using 2 groups to 5 groups with all cubic models to determine the most appropriate number of groups with adequate sample sizes. Subsequently, we tested the best-fit shape of cognitive trajectories. The final models consisted of 3 trajectory groups for each sex, labeled as low, middle, and high based on the value and change in cognitive function scores.

To assess whether the socio-demographic characteristics and health influence factors differed among the 3 cognitive trajectory groups, the chi-square test was employed for categorical variables, while the independent-samples t-test was utilized for continuous variables. Physical performance was an ordinal categorical variable with a score range of 0 to 3, determined by 3 independent measures of 0 or 1 on grip strength, repeated chair stands, and standing balance tests. An ordered logistic regression model was used to analyze the differences in physical performance of participants from the 3 cognitive trajectory groups at baseline, during follow-up, and at the endpoint, using the low trajectory group as the reference. Odds ratios (ORs) and 95% confidence intervals (CIs) of physical performance for each cognitive trajectory group were calculated. Age, marital status, education level, BMI, smoking status, and drinking status were included in adjusted model 1. The presence of chronic diseases, categorized as cardiovascular and cerebrovascular metabolic diseases, psychiatric and neurological diseases, and other diseases, was incorporated into adjusted model 2. Considering that 556 (9.7%) respondents had missing grip strength data due to 1-handed disability or inconvenience, sensitivity analyses were conducted on respondents with complete grip strength data in adjusted model 3 to avoid the manifestation of poor physical performance. All analytical procedures were stratified by sex and residential status. Additionally, we analyzed the relationship between the cognitive trajectory group and each original variable of physical performance to avoid ceiling effects.

All statistical analyses were conducted using Stata version 14.0 (StataCorp., College Station, TX, USA). An association was deemed significant if the associated 2-sided p-value was less than 0.05.

### Ethics statement

The study was approved by the Ethical Review Committee of Peking University (No. IRB00001052-11014), and all participants provided written informed consent.

## RESULTS

### Baseline characteristics of respondents

A total of 5,701 middle-aged and elderly Chinese individuals participated in this study (47.7% male; 21.0% from urban areas), with an average baseline age of 57.8 years (standard deviation, 8.4). Male participants demonstrated higher grip strength than female participants (36.9±8.5 vs. 25.3±6.7 kg, p<0.001) and performed more successful repeated chair stands (86.0 vs. 75.9%, p<0.001). The baseline cognitive function scores were 16.1±4.6 for male participants and 14.3±5.4 for female participants. Demographic characteristics and lifestyle information of the participants at baseline, by sex, are presented in Table 1.

### Characteristics of follow-up assessments by cognitive trajectory group

**Figure 1.**
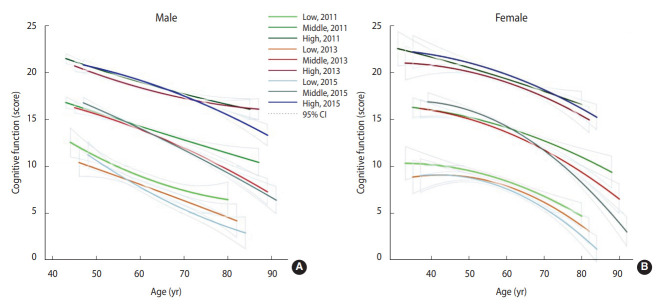
Score distributions for trajectory groups of cognitive function at baseline, follow-up, and endpoint in middle-aged and elderly Chinese participants, 2011-2015 (A: male, B: female). Colored lines indicate the trajectory groups, while grey lines indicate the 95% confidence intervals (CIs) of each group.

The distribution of cognitive function scores for each cognitive trajectory group during the follow-up period is displayed in Figure 1 by sex. According to the best-fit model of group-based trajectory modeling, respondents were divided into 3 groups: 1,104 (19.4%) were in the low group (10.4% male vs. 26.9% female), 2,478 (43.5%) were in the middle group (42.4% male vs. 44.8% female), and 2,119 (37.2%) were in the high group (47.3% male vs. 28.3% female). Overall, the high group performed best in maintaining cognitive function, while the middle group exhibited the greatest cognitive change with age. Female individuals had better cognitive function than male participants at baseline, but they experienced a steeper decline with age.

Table 2 presents the characteristics of the respondents by cognitive trajectory group. Significant differences were observed among the 3 cognitive trajectory groups in terms of sex, residence, marital status, education level, smoking status, alcohol consumption, and BMI. However, no statistical difference in age was observed among the 3 groups. Physical performance at each follow-up differed significantly among the 3 groups (p<0.001), with the group exhibiting higher cognitive function demonstrating better physical performance.

### Differences in physical performance among cognitive trajectory groups

**Figure 2.**
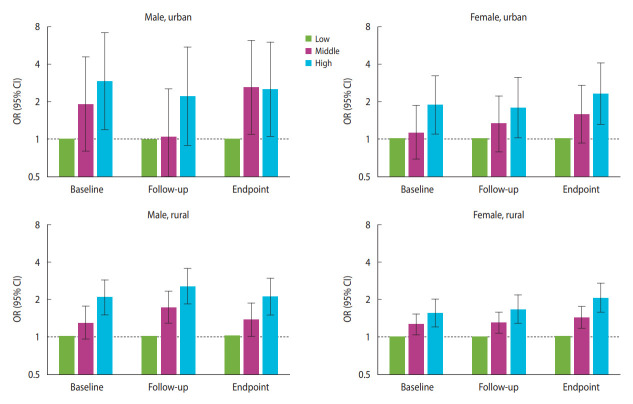
Difference in physical performance of participants from the 3 cognitive trajectory groups, 2011-2015. The low cognitive trajectory group was set as the reference. This model was adjusted for age, marital status, education level, province, drinking and smoking status, body mass index, cardiovascular and cerebrovascular metabolic diseases, neurological and psychiatric disorders, and other diseases. OR, odds ratio; CI, confidence interval.

In the fully adjusted model, compared to the low group, the ORs of better physical performance in the middle and high groups at baseline were as follows: 1.92 (95% CI, 0.80 to 4.57; p=0.142) and 2.92 (95% CI, 1.19 to 7.15; p=0.019) for urban male, 1.12 (95% CI, 0.68 to 1.86; p=0.648) and 1.86 (95% CI, 1.08 to 3.23; p=0.026) for urban female, 1.28 (95% CI, 0.95 to 1.73; p=0.108) and 2.07 (95% CI, 1.48 to 2.88; p<0.001) for rural male, and 1.25 (95% CI, 1.02 to 1.53; p=0.030) and 1.55 (95% CI, 1.18 to 2.02; p<0.001) for rural female. During follow-up, the ORs of better physical performance in the middle and high groups were 1.04 (95% CI, 0.43 to 2.53; p=0.923) and 2.21 (95% CI, 0.89 to 5.50; p=0.089) for urban male, 1.32 (95% CI, 0.78 to 2.21; p=0.297) and 1.77 (95% CI, 1.01 to 3.12; p=0.047) for urban female, 1.71 (95% CI, 1.27 to 2.31; p<0.001) and 2.53 (95% CI, 1.82 to 3.52; p<0.001) for rural male, and 1.30 (95% CI, 1.06 to 1.58; p=0.010) and 1.67 (95% CI, 1.28 to 2.18; p<0.001) for rural female. At the endpoint, the ORs of better physical performance in the middle and high groups were 2.60 (95% CI, 1.09 to 6.22; p=0.032) and 2.53 (95% CI, 1.05 to 6.10; p=0.038) for urban male, 1.57 (95% CI, 0.92 to 2.69; p=0.097) and 2.31 (95% CI, 1.30 to 4.11; p=0.004) for urban female, 1.36 (95% CI, 1.00 to 1.86; p=0.054) and 2.09 (95% CI, 1.48 to 2.95; p<0.001) for rural male, and 1.42 (95% CI, 1.16 to 1.74; p<0.001) and 2.06 (95% CI, 1.57 to 2.71; p<0.001) for rural female (Figure 2, Supplementary Material 1). All trends between the 3 cognitive trajectory groups were statistically significant (p<0.05), except for the urban male group at the endpoint. The crude model and adjusted model 1 are displayed in Supplementary Materials 2 and 3. Upon analyzing each original indicator of physical performance, the trends of grip strength and repeated chair stands between the 3 cognitive trajectory groups were all statistically significant (p<0.05). However, the results of the standing balance test showed no statistical significance (Supplementary Material 4).

### Sensitivity analyses

Sensitivity analyses were performed for respondents with complete grip strength data, separated by sex and residential status (Supplementary Material 5). The results remained consistent with the analysis of all participants.

## DISCUSSION

In this study, we conducted a 4-year follow-up with 5,701 middle-aged and elderly individuals and observed a significant overall decline in cognitive function as they aged. We identified 3 distinct cognitive trajectories, which we labeled as low (19.4%), middle (43.5%), and high (37.2%). All 3 cognitive trajectories displayed parallel downward trends with age, suggesting that cognitive impairment is a chronic and irreversible degenerative pathological process [27]. We discovered that participants with higher cognitive trajectories exhibited better physical performance, and this difference became more pronounced over time. This supports previous research findings that better cognitive function is associated with better physical performance [28,29]. These associations remained significant after adjusting for covariates and in sensitivity analyses.

Our findings demonstrated that participants with better cognitive function at baseline tended to maintain a more stable level of cognitive function as they aged. This trend was consistent with the results of previous regional studies conducted among American and Mexican populations [30,31]. Additionally, higher BMI may be a protective factor against cognitive impairment [32]. Using a nationally representative cohort survey in China, we were the first to report sex differences in cognitive trajectories among an Asian population. We also discovered that while females generally had higher cognitive levels than males, their cognitive function fluctuated and decreased more significantly with age. The results of previous studies are controversial regarding sex differences in cognitive function with age. Although research in American and European populations has indicated that female have higher initial scores on most types of cognitive tests [33,34], no definite result exists regarding whether the rate of decline differs between males and females [33,35,36]. Additional longitudinal studies focusing on Asian populations would help to clarify this pattern [32].

The finding that a decline in overall cognitive function is associated with a decline in physical performance aligns with previous studies [28,29,37]. Prior research has shown that physical performance can predict declines in cognitive function or the risk of mild cognitive impairment and dementia later in life [38,39]. One potential mechanism is that vascular or degenerative damage to the brain, such as that caused by smoking or low hemoglobin level [40,41], may impact both cognitive and motor areas, thus explaining the link between cognitive function and physical performance. Even after adjusting for potential confounding factors, such as smoking status, drinking status, and the presence of multiple chronic diseases, we observed significant correlations between cognitive function and physical performance, particularly in grip strength and repeated stand-up tests.

The differences in physical performance between cognitive trajectory groups also widened among female participants over time, while those in male participants remained stable. However, the underlying mechanism requires further exploration. After stratifying by urban-rural residence, we found that urban participants were more likely to be categorized into the high cognitive trajectory group, while rural participants were more likely to be assigned to the middle-low cognitive groups. This, coupled with the facts that cognitive function declined more rapidly in the lower trajectory groups to which rural participants were more likely to be assigned [42] and that rural participants were more vulnerable due to a lack of education, household wealth, and health insurance [43,44], suggests that greater attention should be paid to slowing down cognitive decline in rural areas.

The present study had several potential limitations. First, although we found that the group with a higher cognitive trajectory exhibited better physical performance, the causal relationship and mechanism underlying this transition remain unknown and warrant further research. Second, while factors such as residence, sex, age, marital status, BMI, smoking, drinking, and chronic diseases were included in our analysis to control for confounding effects, other influential factors such as socioeconomic status and sleep duration could not be incorporated due to the design of the original CHARLS study. Third, approximately 60% of respondents from the original CHARLS dataset had missing values for key information such as cognitive function and grip strength and were therefore excluded from the present analysis. This exclusion may also impact the effect size and generalizability of our findings. Nonetheless, the CHARLS is a database that covers 150 counties/districts and 450 villages/residential committees in 28 provinces of China and possesses strong national representativeness. The relationship between physical performance and cognitive trajectory groups identified in the present study still provides a reliable basis for further research.

Using CHARLS data, we discovered that improved cognitive function was associated with male sex, urban residency, high BMI, and high cognitive trajectory. Participants from the high cognitive trajectory group demonstrated better physical performance, suggesting that early interventions to maintain stable cognitive function may be crucial for preserving optimal physical performance in middle-aged and elderly individuals.

## Figures and Tables

**Table 1.** Baseline characteristics of participants by sex, 2011-2015

Characteristics		Overall	Male	Female	p for difference^1^
No. of participants		5,701 (100)	2,718 (47.7)	2,983 (52.3)	-
	Area of residence, urban	1,195 (21.0)	543 (20.0)	652 (21.9)	0.082
Age (yr)		57.8±8.4	59.3±8.3	56.5±8.2	<0.001
Marital status, married		5,156 (90.4)	2,496 (91.8)	2,660 (89.2)	<0.001
Education level					<0.001
	Illiterate	1,358 (23.8)	294 (10.8)	1,064 (35.7)	-
	Elementary school	2,514 (44.1)	1,289 (47.4)	1,225 (41.1)	-
	≥Middle school	1,829 (32.1)	1,135 (41.8)	694 (23.3)	-
Smoking, yes		2,235 (39.2)	2,039 (75.0)	196 (6.6)	<0.001
Drinking, yes		1,909 (33.5)	1,550 (57.0)	359 (12.0)	<0.001
Body mass index (kg/m^2^)		23.6±3.9	23.1±3.6	24.2±4.0	<0.001
Cardiovascular and cerebrovascular metabolic diseases		2,067 (36.3)	1,021 (37.6)	1,046 (35.1)	0.050
Neurological and psychiatric disorders		149 (2.6)	65 (2.4)	84 (2.8)	0.310
Other diseases		2,825 (49.6)	1,366 (50.3)	1,459 (48.9)	0.310
Physical performance					
	Grip strength (kg)	30.8±9.6	36.9±8.5	25.3±6.7	<0.001
	Repeated chair stands	4,599 (80.7)	2,336 (86.0)	2,263 (75.9)	<0.001
	Standing balance test	5,681 (99.7)	2,708 (99.6)	2,973 (99.7)	0.835
	Cognitive function	15.1±5.1	16.1±4.6	14.3±5.4	<0.001

Values are presented as number (%) or mean±standard deviation.

1 Used to measure the differences between male and female groups.

**Table 2.** Characteristics of participants at baseline, follow-up, and endpoint by cognitive trajectory group, 2011-2015

Variables			Overall	Cognitive trajectory			p for difference^1^
				Low	Middle	High	
No. of participants			5,701 (100)	1,104 (19.4)	2,478 (43.5)	2,119 (37.2)	-
	Sex, male		2,718 (47.7)	287 (26.0)	1,150 (46.4)	1,281 (60.5)	<0.001
	Resident, urban		1,195 (30.0)	115 (10.4)	401 (16.2)	679 (32.0)	<0.001
Baseline							
Age (yr)			57.8±8.4	57.9±7.9	57.6±8.6	58.0±8.4	0.276
	Marital status, married		5,156 (90.4)	954 (86.4)	2,236 (90.2)	1,966 (92.8)	<0.001
	Education level						<0.001
		Illiterate	1,358 (23.8)	689 (62.4)	587 (23.7)	82 (3.9)	-
		Elementary school	2,514 (44.1)	365 (33.1)	1,276 (51.5)	873 (41.2)	-
		≥Middle school	1,829 (32.1)	50 (4.5)	615 (24.8)	1,164 (54.9)	-
	Smoking, yes		2,235 (39.2)	294 (26.6)	961 (38.8)	980 (46.3)	<0.001
	Drinking, yes		1,909 (33.5)	251 (22.7)	786 (31.7)	872 (41.2)	<0.001
	Body mass index (kg/m^2^)		23.63±3.85	23.16±3.77	23.56±3.90	23.96±3.81	<0.001
	Physical performance		2.30±0.69	2.03±0.68	2.26±0.69	2.47±0.64	<0.001
	Cognitive function		15.13±5.15	8.71±3.60	14.39±3.53	19.35±3.19	<0.001
Follow-up							
	Physical performance		2.30±0.69	2.02±0.69	2.28±0.69	2.48±0.64	<0.001
	Cognitive function		14.32±5.18	7.70±3.56	13.63±3.46	18.59±3.16	<0.001
Endpoint							
	Physical performance		2.31±0.68	2.03±0.70	2.29±0.67	2.49±0.62	<0.001
	Cognitive function		14.32±5.58	7.02±3.66	13.56±3.74	18.95±3.28	<0.001

Values are presented as number (%) or mean±standard deviation.

1 Used to measure the differences between the 3 cognitive trajectory groups.

## SUPPLEMENTARY MATERIALS

Supplement Material 1. Difference in physical performance of participants from three cognitive trajectory groups(crude model) (OR and 95% CI): 2011-2015

Supplement Material 2. Difference in physical performance of participants from three cognitive trajectory groups(adjusted model 1): (OR and 95% CI): 2011-2015

Supplement Material 3. Difference in physical performance of participants from three cognitive trajectory groups(adjusted model 2): (OR and 95% CI): 2011-2015

Supplement Material 4. Difference in physical performance of participants with complete grip strength data(adjusted model 3) (N=5145, OR and 95% CI)

Supplement Material 5. Linear regression model for each original indicators of physical performance from three cognitive trajectory groups: (β/OR and 95% CI): 2011-2015
