# Supplementary material for: Exploring cognitive trajectories and their association with physical performance: evidence from the China Health and Retirement Longitudinal Study
Source: Epidemiol Health. 2023 Jul 9;45:e2023064. doi: 10.4178/epih.e2023064 (PMC10667582; doi:10.4178/epih.e2023064)
Supplement: Supplement Material 1. — Difference in physical performance of participants from three cognitive trajectory groups(crude model) (OR and 95% CI): 2011-2015 [file epih-45-e2023064-Supplementary-1.docx]

**Supplementary Material 1. Difference in physical performance of participants from three cognitive trajectory groups(crude model) (OR and 95% CI): 2011-2015**

| Cognition trajectory group | | Total | | |  | Male | | |  | Female | | | |
| --- | --- | --- | --- | --- | --- | --- | --- | --- | --- | --- | --- | --- | --- |
|  |  | Baseline | Follow-up | Endpoint |  | Baseline | Follow-up | Endpoint |  | Baseline | Follow-up | Endpoint |  |
| Total | Low | 1(Reference) | 1(Reference) | 1(Reference) |  | 1(Reference) | 1(Reference) | 1(Reference) |  | 1(Reference) | 1(Reference) | 1(Reference) |  |
|  | Middle | 1.91 (1.67-2.19)^***^ | 2.01 (1.76-2.30)^***^ | 2.08 (1.82-2.39)^***^ |  | 1.47 (1.13-1.91)^**^ | 1.63 (1.26-2.12)^***^ | 1.50 (1.15-1.95)^**^ |  | 1.36 (1.14-1.62)^***^ | 1.41 (1.18-1.67)^***^ | 1.54 (1.29-1.84)^***^ |  |
|  | High | 3.52 (3.05-4.05)^***^ | 3.69 (3.20-4.25)^***^ | 3.83 (3.32-4.42)^***^ |  | 2.30 (1.77-2.99)^***^ | 2.58 (1.98-3.35)^***^ | 2.20 (1.69-2.88)^***^ |  | 1.98 (1.63-2.40)^***^ | 2.04 (1.68-2.47)^***^ | 2.44 (2.00-2.97)^***^ |  |
|  | *P*_trend_ | <0.001 | <0.001 | <0.001 |  | <0.001 | <0.001 | <0.001 |  | <0.001 | <0.001 | <0.001 |  |
| Urban | Low | 1(Reference) | 1(Reference) | 1(Reference) |  | 1(Reference) | 1(Reference) | 1(Reference) |  | 1(Reference) | 1(Reference) | 1(Reference) |  |
|  | Middle | 1.94 (1.32-2.85)^***^ | 1.62 (1.09-2.40)^*^ | 2.42 (1.62-3.61)^***^ |  | 2.19 (0.96-4.97) | 1.25 (0.54-2.88) | 3.54 (1.59-7.89)^**^ |  | 1.35 (0.85-2.15) | 1.39 (0.86-2.26) | 1.76 (1.07-2.89)^*^ |  |
|  | High | 3.40 (2.34-4.93)^***^ | 2.82 (1.92-4.12)^***^ | 3.85 (2.61-5.67)^***^ |  | 2.78 (1.27-6.09)^*^ | 2.43 (1.09-5.45)^*^ | 3.85 (1.82-8.17)^***^ |  | 2.12 (1.34-3.34)^***^ | 1.66 (1.04-2.67)^*^ | 2.40 (1.48-3.90)^***^ |  |
|  | *P*_trend_ | <0.001 | <0.001 | <0.001 |  | 0.017 | 0.001 | 0.008 |  | <0.001 | 0.033 | <0.001 |  |
| Rural | Low | 1(Reference) | 1(Reference) | 1(Reference) |  | 1(Reference) | 1(Reference) | 1(Reference) |  | 1(Reference) | 1(Reference) | 1(Reference) |  |
|  | Middle | 1.93 (1.67-2.24)^***^ | 2.05 (1.77-2.37)^***^ | 2.03 (1.76-2.35)^***^ |  | 1.39 (1.06-1.83)^*^ | 1.66 (1.27-2.19)^***^ | 1.34 (1.01-1.77)* |  | 1.39 (1.15-1.68)^***^ | 1.37 (1.13-1.65)^***^ | 1.49 (1.23-1.80)^***^ |  |
|  | High | 3.71 (3.16-4.35)^***^ | 3.77 (3.22-4.42)^***^ | 3.94 (3.36-4.62)^***^ |  | 2.22 (1.67-2.94)^***^ | 2.39 (1.80-3.17)^***^ | 2.00 (1.50-2.67)^***^ |  | 1.96 (1.56-2.47)^***^ | 1.96 (1.56-2.46)^***^ | 2.41 (1.92-3.04)^***^ |  |
|  | *P*_trend_ | <0.001 | <0.001 | <0.001 |  | <0.001 | <0.001 | <0.001 |  | <0.001 | <0.001 | <0.001 |  |

Note. CI = Confidence Interval; OR=Odds Ratio; ^***^*p*<0.001; ^**^*p*<0.01; ^*^*p*<0.05

^a^The low trajectory group was set as the reference.

^b^P for trend measures whether the linear tendency is significant between the three cognitive trajectory groups.
