# Supplementary material for: Exploring cognitive trajectories and their association with physical performance: evidence from the China Health and Retirement Longitudinal Study
Source: Epidemiol Health. 2023 Jul 9;45:e2023064. doi: 10.4178/epih.e2023064 (PMC10667582; doi:10.4178/epih.e2023064)
Supplement: Supplement Material 3. — Difference in physical performance of participants from three cognitive trajectory groups(adjusted model 2): (OR and 95% CI): 2011-2015 [file epih-45-e2023064-Supplementary-3.docx]

**Supplementary Material 3. Difference in physical performance of participants from three cognitive trajectory groups(adjusted model 2): (OR and 95% CI): 2011-2015**

| Cognition trajectory group | | Total | | |  | Male | | |  | Female | | | |
| --- | --- | --- | --- | --- | --- | --- | --- | --- | --- | --- | --- | --- | --- |
|  |  | Baseline | Follow-up | Endpoint |  | Baseline | Follow-up | Endpoint |  | Baseline | Follow-up | Endpoint |  |
| Total | Low | 1(Reference) | 1(Reference) | 1(Reference) |  | 1(Reference) | 1(Reference) | 1(Reference) |  | 1(Reference) | 1(Reference) | 1(Reference) |  |
|  | Middle | 1.23 (1.06-1.43)^**^ | 1.37 (1.17-1.59)^***^ | 1.40 (1.20-1.64)^***^ |  | 1.33 (1.00-1.77) ^*^ | 1.59 (1.20-2.11)^***^ | 1.41 (1.05-1.85)^*^ |  | 1.22 (1.01-1.47)^*^ | 1.31 (1.09-1.58)^**^ | 1.44 (1.19-1.74)^***^ |  |
|  | High | 1.79 (1.49-2.15) ^***^ | 1.94 (1.61-2.32) ^***^ | 2.04 (1.69-2.45) ^***^ |  | 2.12 (1.55-2.89) ^**^^*^ | 2.49 (1.83-3.40) ^***^ | 2.06 (1.50-2.84) ^***^ |  | 1.64 (1.29-2.08) ^***^ | 1.68 (1.33-2.13) ^***^ | 2.09 (1.64-2.66) ^***^ |  |
|  | *P*_trend_ | <0.001 | <0.001 | <0.001 |  | <0.001 | <0.001 | <0.001 |  | <0.001 | <0.001 | <0.001 |  |
| Urban | Low | 1(Reference) | 1(Reference) | 1(Reference) |  | 1(Reference) | 1(Reference) | 1(Reference) |  | 1(Reference) | 1(Reference) | 1(Reference) |  |
|  | Middle | 1.32 (0.86-2.04) | 1.19 (0.76-1.86) | 1.76 (1.12-2.76) ^*^ |  | 1.92 (0.80-4.57) | 1.04 (0.43-2.53) | 2.60 (1.09-6.22) ^*^ |  | 1.12 (0.68-1.86) | 1.32 (0.78-2.21) | 1.57(0.92-2.69) |  |
|  | High | 2.07 (1.30-3.38) ^**^ | 1.84 (1.14-3.98) ^**^ | 2.25 (1.39-3.62) ^***^ |  | 2.92 (1.19-7.15) ^*^ | 2.21 (0.89-5.50) | 2.53 (1.05-6.10) ^*^ |  | 1.86 (1.08-3.23) ^*^ | 1.77 (1.01-3.12) ^*^ | 2.31 (1.30-4.11) ^**^ |  |
|  | *P*_trend_ | <0.001 | 0.002 | 0.002 |  | 0.013 | 0.006 | 0.166 |  | 0.006 | 0.032 | 0.003 |  |
| Rural | Low | 1(Reference) | 1(Reference) | 1(Reference) |  | 1(Reference) | 1(Reference) | 1(Reference) |  | 1(Reference) | 1(Reference) | 1(Reference) |  |
|  | Middle | 1.23 (1.04-1.45) ^*^ | 1.39 (1.18-1.64) ^***^ | 1.38 (1.17-1.63) ^***^ |  | 1.28 (0.95-1.73) | 1.71 (1.27-2.31) ^***^ | 1.36 (1.00-1.86) |  | 1.25 (1.02-1.53) ^*^ | 1.30 (1.06-1.58) ^*^ | 1.42 (1.16-1.74) ^***^ |  |
|  | High | 1.74 (1.42-2.12) ^***^ | 1.92 (1.58-2.35) ^***^ | 2.05 (1.67-2.51) ^***^ |  | 2.07 (1.48-2.88) ^***^ | 2.53 (1.82-3.52) ^***^ | 2.09 (1.48-2.95) ^***^ |  | 1.55 (1.18-2.02) ^*^^**^ | 1.67 (1.28-2.18) ^***^ | 2.06 (1.57-2.71) ^***^ |  |
|  | *P*_trend_ | <0.001 | <0.001 | <0.001 |  | <0.001 | <0.001 | <0.001 |  | <0.001 | <0.001 | <0.001 |  |

Note. CI = Confidence Interval; OR=Odds Ratio; ^***^*p*<0.001; ^**^*p*<0.01; ^*^*p*<0.05; Adjusted model 2 was adjusted for age, marital status, education level, province, drinking and smoking status, body mass index(BMI), Cardiovascular and cerebrovascular metabolic diseases, neurological and psychiatric disorders and other diseases. Residence was adjusted in sex stratified analysis. Sex was adjusted in residence stratified analysis. Sex and residence were adjusted in overall analysis.

^a^The low trajectory group was set as the reference.

^b^P for trend measures whether the linear tendency is significant between the three cognitive trajectory groups.
