# Supplementary material for: Exploring cognitive trajectories and their association with physical performance: evidence from the China Health and Retirement Longitudinal Study
Source: Epidemiol Health. 2023 Jul 9;45:e2023064. doi: 10.4178/epih.e2023064 (PMC10667582; doi:10.4178/epih.e2023064)
Supplement: Supplement Material 4. — Difference in physical performance of participants with complete grip strength data(adjusted model 3) (N=5145, OR and 95% CI) [file epih-45-e2023064-Supplementary-4.docx]

**Supplementary Material 4. Difference in physical performance of participants with complete grip strength data(adjusted model 3) (N=5145, OR and 95% CI)**

| Cognition trajectory group | | Total | | |  | Male | | |  | Female | | | |
| --- | --- | --- | --- | --- | --- | --- | --- | --- | --- | --- | --- | --- | --- |
|  |  | Baseline | Follow-up | Endpoint |  | Baseline | Follow-up | Endpoint |  | Baseline | Follow-up | Endpoint |  |
| Total | Low | 1(Reference) | 1(Reference) | 1(Reference) |  | 1(Reference) | 1(Reference) | 1(Reference) |  | 1(Reference) | 1(Reference) | 1(Reference) |  |
|  | Middle | 1.22 (1.04-1.44) ^*^ | 1.37 (1.16-1.61) ^***^ | 1.40 (1.18-1.65) ^***^ |  | 1.38 (1.02-1.87)^*^ | 1.67 (1.24-2.25)^***^ | 1.47 (1.07-2.00)^*^ |  | 1.19 (0.98-1.44) | 1.29 (1.06-1.57)^*^ | 1.41 (1.15-1.72)^***^ |  |
|  | High | 1.78 (1.47-2.16) ^***^ | 1.92 (1.59-2.33) ^***^ | 2.04 (1.67-2.48) ^***^ |  | 2.23 (1.60-3.10)^***^ | 2.71 (1.95-3.75)^***^ | 2.14 (1.52-3.00)^***^ |  | 1.59 (1.24-2.04)^***^ | 1.57 (1.23-2.02)^***^ | 2.06 (1.59-2.66)^***^ |  |
|  | *P*_trend_ | <0.001 | <0.001 | <0.001 |  | <0.001 | <0.001 | <0.001 |  | <0.001 | <0.001 | <0.001 |  |
| Urban | Low | 1(Reference) | 1(Reference) | 1(Reference) |  | 1(Reference) | 1(Reference) | 1(Reference) |  | 1(Reference) | 1(Reference) | 1(Reference) |  |
|  | Middle | 1.28 (0.81-2.03) | 1.15 (0.72-1.86) | 1.76 (1.09-2.84)^*^ |  | 2.04 (0.82-5.06) | 1.04 (0.41-2.66) | 2.28 (0.88-5.86) |  | 1.10 (0.65-1.88) | 1.29 (0.74-2.24) | 1.61 (0.91-2.84) |  |
|  | High | 2.05 (1.25-3.36)^**^ | 1.65 (0.99-2.76) | 2.18 (1.31-3.62)^**^ |  | 3.14 (1.23-7.99)^*^ | 2.35 (0.89-6.19) | 2.02 (0.78-5.23) |  | 1.85 (1.02-3.34) ^*^ | 1.48 (0.80-2.73) | 2.34 (1.27-4.34)^*^^*^ |  |
|  | *P*_trend_ | <0.001 | 0.015 | 0.005 |  | 0.012 | 0.004 | 0.459 |  | 0.010 | 0.224 | 0.005 |  |
| Rural | Low | 1(Reference) | 1(Reference) | 1(Reference) |  | 1(Reference) | 1(Reference) | 1(Reference) |  | 1(Reference) | 1(Reference) | 1(Reference) |  |
|  | Middle | 1.23 (1.03-1.46)^*^ | 1.40 (1.18-1.66)^***^ | 1.38 (1.15-1.64)^***^ |  | 1.34 (0.97-1.84) | 1.83 (1.33-2.51)^***^ | 1.45 (1.04-2.02)^*^ |  | 1.22 (0.99-1.51) | 1.28 (1.04-1.58)^*^ | 1.38 (1.12-1.72)^**^ |  |
|  | High | 1.72 (1.40-2.13)^***^ | 1.95 (1.58-2.40)^***^ | 2.06 (1.66-2.56)^***^ |  | 2.18 (1.53-3.11)^***^ | 2.77 (1.95-3.92)^***^ | 2.26 (1.56-3.27)^***^ |  | 1.49 (1.12-1.97)^**^ | 1.62 (1.23-2.13)^***^ | 2.01 (1.51-2.68)^***^ |  |
|  | *P*_trend_ | <0.001 | <0.001 | <0.001 |  | <0.001 | <0.001 | <0.001 |  | 0.005 | 0.001 | <0.001 |  |

Note. CI = Confidence Interval; OR=Odds Ratio; ^***^*p*<0.001; ^**^*p*<0.01; ^*^*p*<0.05; This model were adjusted for age, marital status, education level, province, drinking and smoking status, body mass index(BMI). Residence was adjusted in sex stratified analysis. Sex was adjusted in residence stratified analysis. Sex and residence were adjusted in overall analysis.

^a^The low trajectory group was set as the reference.

^b^P for trend measures whether the linear tendency is significant between the three cognitive trajectory groups.
