# Supplementary material for: Exploring cognitive trajectories and their association with physical performance: evidence from the China Health and Retirement Longitudinal Study
Source: Epidemiol Health. 2023 Jul 9;45:e2023064. doi: 10.4178/epih.e2023064 (PMC10667582; doi:10.4178/epih.e2023064)
Supplement: Supplement Material 5. — Linear regression model for each original indicators of physical performance from three cognitive trajectory groups: (β/OR and 95% CI): 2011-2015 [file epih-45-e2023064-Supplementary-5.docx]

**Supplementary Material 5. Linear regression model for each original** **indicators of physical performance** **from three cognitive trajectory groups: (β/OR and 95% CI): 2011-2015**

| Physical performance | Cognition trajectory group | Baseline | Follow-up | Endpoint |
| --- | --- | --- | --- | --- |
| Grip strength  β (95%CI) | Low | 1(Reference) | 1(Reference) | 1(Reference) |
|  | Middle | 0.63 (0.11-1.15)* | 0.89 (0.39-1.39)*** | 0.71 (0.26-1.17)** |
|  | High | 2.06 (1.45-2.67)*** | 2.03 (1.45-2.62)*** | 2.38 (1.85-2.91)*** |
|  | P_trend_ | <0.001 | <0.001 | <0.001 |
| Repeated chair stands  OR (95%CI) | Low | 1(Reference) | 1(Reference) | 1(Reference) |
|  | Middle | 1.29 (1.07-1.54)** | 1.42 (1.18-1.70)*** | 1.37 (1.14-1.64)*** |
|  | High | 1.90 (1.52-2.37)*** | 1.92 (1.54-2.40)*** | 1.81 (1.45-2.27)*** |
|  | P_trend_ | <0.001 | <0.001 | <0.001 |
| Standing balance test  OR (95%CI) | Low | 1(Reference) | 1(Reference) | 1(Reference) |
|  | Middle | 0.46 (0.10-2.17) | 0.70 (0.21-2.29) | 1.61 (0.46-5.62) |
|  | High | 1.01 (0.17-6.09) | 0.77 (0.19-3.17) | 1.68 (0.25-11.14) |
|  | P_trend_ | 0.601 | 0.770 | 0.491‘ |

Note. CI = Confidence Interval; OR=Odds Ratio; β= Regression coefficients; ^***^*p*<0.001; ^**^*p*<0.01; ^*^*p*<0.05; This model was adjusted for sex, residence, age, marital status, education level, province, drinking and smoking status, body mass index(BMI).

^a^The low trajectory group was set as the reference.

^b^P for trend measures whether the linear tendency is significant between the three cognitive trajectory groups.
